# p73: a Positive or Negative Regulator of Angiogenesis, or Both?

**DOI:** 10.1128/MCB.00929-15

**Published:** 2016-03-01

**Authors:** Kanaga Sabapathy

**Affiliations:** Division of Cellular and Molecular Research, Humphrey Oei Institute of Cancer Research, National Cancer Centre Singapore, Singapore, Republic of Singapore; Cancer and Stem Cell Biology Program, Duke-NUS Graduate Medical School, Singapore, Republic of Singapore; Institute of Molecular and Cell Biology, Biopolis, Singapore, Republic of Singapore; Department of Biochemistry, National University of Singapore, Singapore, Republic of Singapore

## Abstract

The role of p73, the homologue of the tumor suppressor p53, in regulating angiogenesis has recently been extensively investigated, resulting in the publication of five articles. Of these, two studies suggested a suppressive role, while the others implied a stimulatory role for the p73 isoforms in regulating angiogenesis. A negative role for TAp73, the full-length form that is often associated with tumor suppression, in blood vessel formation, is consistent with its general attributes and was proposed to be effected indirectly through the degradation of hypoxia-inducible factor 1α (HIF1-α), the master angiogenic regulator. In contrast, a positive role for TAp73 coincides with its recently understood role in supporting cellular survival and thus tumorigenesis, consistent with TAp73 being not-mutated but rather often overexpressed in clinical contexts. In the latter case, TAp73 expression was induced by hypoxia via HIF1-α, and it appears to directly promote angiogenic target gene activation and blood vessel formation independent of HIF1-α. This mini review will provide an overview of these seemingly opposite recent findings as well as earlier data, which collectively establish the definite possibility that TAp73 is indeed capable of both promoting and inhibiting angiogenesis, depending on the cellular context.

## p73 AND ANGIOGENESIS: OVERVIEW FROM THE RECENT WORK

p73 is the homologue of the master tumor suppressor p53 and has been shown to exist in two major forms. Of these, TAp73 is the full-length version and is akin to p53 in its functionality based on cell-based assays. On the other hand, DNp73 is an amino-terminally truncated version that lacks the transactivation domain and, thus, has been suggested to be an antiapoptotic and prosurvival protein, by virtue of its ability to bind and inhibit both the TAp73 and p53 forms (1, 2). Initial knockout of all the p73 forms did not result in an increased propensity for tumor development (3). However, subsequent isoform-specific knockouts suggested that the absence of TAp73 does lead to spontaneous tumor formation, albeit in a significantly delayed manner, insinuating that while TAp73 has tumor suppressive properties, they are much weaker than those of its counterpart p53 (4, 5). In contrast, DNp73 knockout mice were not tumor prone as expected, and cells from these mice were more sensitive to p53-dependent death (6). Moreover, all these mice displayed multiple phenotypes in neuronal development, immune response, etc. (3, 4, 6), implying a role for both the p73 proteins beyond tumor suppression. Interestingly, human clinical data indicate that *p73* is hardly mutated but both the p73 isoforms are overexpressed to various extents in several cancers (7, 8). These data intimate a role for both these forms in tumor promotion, which has been supported by recent findings on TAp73's ability to regulate several prosurvival pathways (9, 10).

It is against this backdrop that the role of the p73 proteins in angiogenesis was investigated. Five recent publications have evaluated the role of both the TAp73 and DNp73 forms in various contexts, all using genetically modified systems lacking the proteins, as well as through silencing or overexpression experiments (11–15). Among these, two reports suggest that TAp73 has a suppressive effect on angiogenesis, with one of them indicating that DNp73 is also proangiogenic (11, 12). Conversely, the other three reports suggest that both the TAp73 and DNp73 forms are proangiogenic (13–15). One common conclusion of all these studies is that DNp73 is a proangiogenic protein whose absence or overexpression markedly affects blood vessel formation. However, the role of TAp73 in angiogenesis appears murky from these reports.

The antiangiogenic theory of TAp73 suggests that there is increased angiogenesis in the absence of TAp73, based on data obtained from three models: xenograft models of mouse embryonic fibroblasts (MEFs) transformed with E1A/ras, lymphomas originating from Eμ-myc transgenic mice, and finally, a model of 12-*O*-tetradecanoylphorbol-13-acetate (TPA)–7,12-dimethylbenz[a]anthracene (DMBA)-induced tumor formation (Table 1) (11, 12). Furthermore, overexpression of TAp73 led to decreased angiogenesis when tumor growth was inhibited in a human xenograft model, and the opposite results were obtained when TAp73 was silenced. Moreover, *ex vivo* culturing of aorta ring samples from TAp73^−/−^ mice led to increased new blood vessel formation (11). In addition, these authors showed increased angiogenic target gene activation and angiogenesis upon TAp73 silencing, which was consistent with the latter's negative regulatory effects on hypoxia-inducible factor 1α (HIF1-α) levels (11). A role for TAp73 in facilitating interaction and subsequent regulation of HIF1-α levels by the E3 ubiquitin ligase MDM2 was proposed (11). Furthermore, evidence was provided that HIF1-α was indeed essential to enact TAp73's negative role on angiogenesis, as silencing of HIF1-α led to the reversal of the angiogenic target gene expression (12). Collectively, the data from these studies suggest a model in which TAp73 negatively regulates HIF1-α expression through MDM2-mediated degradation, which results in an indirect antiangiogenic phenotype.

**TABLE 1:** Summary of results from different studies on TAp73's role in angiogenesis^*a*^

Model system	Reference	Effect on tumor size	Effect on angiogenesis	*Vegf-A*/angiogenic gene expression		Conditions for gene silencing/inducible overexpression	p53 status
				*In vivo*	*In vitro*		
Tumor models							
TPA-DMBA chemical carcinogenesis in TAp73^−/−^ mice	**11**	**Larger tumors**	**Increased**				
Eμ-Myc transgenic mouse lymphomagenesis in TAp73^−/−^ mice	**12**		**Increased**				
E1a/Ras-transformed TAp73^−/−^ MEFs in nude/Scid mice	**12**	**Larger tumors**	**Increased**	**Increased**	**Increased**		
	13	Smaller tumors	Decreased		Decreased		Wild type
Xenograft model in nude mice with H1299 cells with TAp73 silencing	**11**	**Larger tumors**	**Increased**	**Increased**	**Increased**^***b***^	**Stable**	**Null**
	13				Decreased	Transient	Null
Xenograft model in nude/Scid mice with H1299/SAOS2 cells with inducible TAp73 expression	**11**	**Smaller tumors (SAOS2)**			**Decreased (H1299)**	**Long term, from initiation of tumors (5 weeks)**	**Null**
	**13**	No difference (H1299 and SAOS2)	Increased		Increased (SAOS2)	Transient (6 days after tumor establishment)	Null
Other models							
Aorta ring from TAp73^−/−^ mice	**11**		**Increased**				
Retina and iPSC from total p73^−/−^ mice and MSC with p73DD	15		Decreased in all cases	Decreased in retina	Decreased in MSC		
HUVEC cells with p73DD overexpression or total p73 or TAp73 silencing	15		Decreased (p73DD and total p73 silenced) and no difference (TAp73 silenced)		Decreased in all cases	Transient	Wild type

a Data from articles that demonstrate an inhibitory role for TAp73 in angiogenesis are in boldface. Data from reports on TAp73 as a positive regulator of angiogenesis are in lightface. A cell without data represents data not provided in the article.

b From reference 12.

On the other hand, the proponents of TAp73 as a positive regulator of angiogenesis demonstrated that TAp73 is stabilized by hypoxia, a physiological stimulus for angiogenesis (16), via HIF1-α-mediated suppression of the E3 ligase SIAH1 (13). TAp73 stabilization resulted in the direct activation of a variety of angiogenic target genes by TAp73, including *Vegf-A*, in a manner independent of HIF1-α. A similar scenario was also proposed for DNp73 (14). TAp73 (and DNp73) binds to the angiogenic target gene regulatory sites that contain SP1 regions, but this does not require the HIF1-α binding hypoxia response elements (HREs) (13). The absence of TAp73 (or similarly, DNp73) thus led to reduced angiogenic target gene activation, whereas its transient overexpression led to the induction of a variety of these target genes. Consistently, tumor size and blood vessel density correlated significantly with TAp73 expression in xenograft models utilizing either E1A/Ras-transformed TAp73 knockout MEFs or TAp73-inducible human tumor cell lines (Table 1). In the third paper that supports a role for p73 in angiogenesis, the authors utilized total-p73-knockout cells (lacking both TAp73 and DNp73), analyzing induced pluripotent cells (iPSC) and embryonic stem cells (MSC), both of which showed reduced angiogenesis and sprouting of vessels (15). Similarly, vascular development in the mouse retina was also perturbed due to p73 deficiency. Moreover, a dominant-negative p73DD form compromised endothelial differentiation and angiogenesis. Using human umbilical vein endothelial cells (HUVEC), these authors additionally showed that DNp73 has a more significant role than TAp73 in regulating *Vegf-A* expression, and silencing DNp73 had a more dramatic effect on tube morphogenesis and migration (15). Together, these data suggest that TAp73 and DNp73 are both proangiogenic, though there may be differences in the extent and contexts of their activation and promotion of angiogenesis.

Collectively, these studies demonstrate that TAp73 can regulate angiogenesis in both directions, though the basis for the manifestation of these opposite outcomes is unclear. One possibility is that the systems used in the above-described studies have distinct variables that could provide some insights into the deterministic factors, which are discussed in this minireview. Alternatively, the effects of TAp73 on angiogenesis may occur sequentially, in distinct temporal realms, thereby establishing a potential regulatory loop, or under different spatiotemporal conditions that provide a context for the selective manifestation of these phenotypes. This concept has also been developed into a bifunctional model to explain all the recent findings.

## ARE THE OPPOSITE EFFECTS OF TAp73 ON ANGIOGENESIS A REFLECTION OF ITS TUMOR REGULATORY PROPERTIES?

The differential effects of TAp73 on angiogenesis are possibly due to the state and context of TAp73 activation. Interestingly, overexpression of TAp73 had opposite outcomes in the recent studies, and Table 1 summarizes the differences and similarities in parameters used in them. In one case, short-term induction of TAp73 for 6 days in well-developed tumors led, prior to any effect on tumor volume and apoptosis, to increased angiogenesis *in vivo* (13). In the other case, long-term TAp73 induction for 5 weeks from the initiation of tumors led to significant retardation of tumor growth and resulted in inhibition of angiogenesis (11). Likewise, in one case, transient silencing of p73 expression led to decreased angiogenic target gene activation in H1299 cells (13), but in another case, where TAp73 was stably silenced, the opposite effect was observed (11). While these data seem distinctly opposite with the use of similar cellular systems, it is worth considering that the temporal effects or the intensity of TAp73 activation may be deterministic of the outcome. For instance, the transient *in vitro* and *in vivo* overexpression of TAp73 led to proangiogenic targets being turned on, prior to the manifestation of tumor-suppressive effects (13). In contrast, long-term induction of TAp73 that led to growth inhibition also led to angiogenic suppression (11). Similarly, the opposing outcomes due to silencing TAp73 could also be attributed to the levels and/or length of silencing. These opposite outcomes on angiogenesis thus mirror TAp73's effects on growth: promotion or inhibition, with the latter scenario appearing to have a consequential effect on cell fate. This is reminiscent of the effects of p53 on *Vegf-A* expression. In the initial hypoxic phase, p53 led to *Vegf-A* activation, whereas long-term continued hypoxia led to its suppression, going along with the tumor-suppressive phenotype in the latter case (17). Whatever the scenario might be, this issue requires further investigation to understand whether the exquisite regulation of TAp73 may be responsible for the differing angiogenesis phenotypes, as it is for cellular growth and survival.

## TAp73 AND *Vegf-A* REGULATION

While the absence or overexpression of TAp73 has clearly been shown in the above-described reports to regulate the expression of angiogenic genes differently, including the prototype *Vegf-A*, other data also exist that have investigated this phenomenon. In these earlier reports, transient-transfection assays were employed to evaluate the role of TAp73 on *Vegf-A* regulation, either by using *Vegf-A* promoter-luciferase reporter constructs or by analyzing the effects on endogenous *Vegf-A* expression (18, 19). Remarkably, these studies are also divided, supporting both an activating and an inhibitory effect of TAp73 on *Vegf-A* expression. Salimath et al. suggested that overexpression of TAp73α led to decreased *Vegf-A* expression, concomitant with increased *p21* expression in two cell lines (18). Detailed investigation using *Vegf-A* promoter deletion constructs led to the identification of a 35-bp element (−85 to −50) that was responsible for TAp73-mediated suppression. This region contains an SP1 site that, when mutated, was unable to confer on TAp73 the ability to suppress the promoter region. In another study, Vikhanskaya et al. demonstrated that TAp73α was able to induce the expression of *Vegf-A*, using either stable or transient TAp73α expression in a variety of tumor cell lines (19). *Vegf-A* promoter deletion analyses led to the identification of a 1,005-bp region that conferred this positive regulation, which was independent of HIF1-α. In the recent reports demonstrating a suppressive role for TAp73 on angiogenesis, TAp73's ability to negatively regulate HIF1-α stability was shown to lead to *Vegf-A* regulation (11, 12), thus implying a direct role for the HREs in this process. On the other hand, the studies supporting a positive role for TAp73 in regulating angiogenesis showed it to be directly bound to a region between −161 and +44 of the *Vegf-A* promoter that contains SP1 sites and, more importantly, lacks any HREs, to regulate *Vegf-A* expression independently of HIF1-α (13).

What is evident from these data is that TAp73 appears to have the ability to regulate *Vegf-A* expression either way, by directly binding to the promoter or indirectly. This is reminiscent of p53's role in regulating *Vegf-A* expression, which was similarly shown to work both ways and which appears to occur in a temporal fashion (20).

## p73 AND Vegf-A EXPRESSION IN CLINICAL SAMPLES

Earlier studies have also tried to establish a correlation between the expression of p73 and Vegf-A using clinical material. The initial data analyzing 56 colorectal samples suggested a positive correlation between p73 and Vegf-A expression by immunohistochemical (IHC) analyses (21). Subsequently, a second group of 112 colorectal patient samples were used to analyze the transcript levels of *TAp73*, *DNp73*, and *Vegf-A* (22). This work also uncovered a positive correlation between both *TAp73* and *DNp73* expression and *Vegf-A* expression. In the recent studies that evaluated the role of TAp73 in angiogenesis, IHC analysis of colorectal and breast cancer tissue microarrays also showed a positive correlation between TAp73 and Vegf-A expression (13). Similar results were obtained with transcriptomic analysis of a large panel of angiogenic genes in gastric cancer data sets (13), strongly supporting the idea of a positive role for TAp73 in regulating angiogenesis in cancers. However, a negative correlation between p73 and HIF1-α activity and angiogenesis in lung cancer samples was noted by transcriptomic analysis in the other study (11). Thus, although a larger number of studies show a direct positive correlation between TAp73 and Vegf-A expression in various tumor types, opposite data also exist to support the other possibility of negative regulation of *Vegf-A* by TAp73.

## IS THERE A BASIS FOR THE DIFFERENTIAL REGULATION OF *Vegf-A* BY TAp73?

While the determinants of how and when TAp73 positively or negatively regulates *Vegf-A* expression are unclear, early data suggested a possibility that is worth revisiting. In their analyses of the *Vegf-A* promoter, Vikhanskaya et al. noted that TAp73 was able to positively regulate *Vegf-A* in cells that had wild-type p53 (19). They alluded to the fact that this was not the case in tumor cell lines with mutant p53, where there was an apparent negative regulation. Consistent with these findings, the original study that reported a suppressive role for TAp73 in *Vegf-A* expression had used two cell lines that led to this conclusion: of these, one lacked p53 expression (SAOS2) and the other had an inactivated p53 (A293) (18), suggesting that the status of p53 might be a determinant for the apparently contrasting activity of TAp73 in regulating *Vegf-A* expression and, thus, by implication, angiogenesis. One cannot exclude the possibilities that some of the cells used in the recent studies had the *p53* gene mutated, given that *p53* is readily mutated in MEFs in culture (23). The studies suggesting the opposite roles for TAp73 in angiogenesis used the same TAp73^−/−^ MEFs that have been transformed with E1a/Ras (12, 13). Also, the same cells have been used by other investigators to demonstrate a positive role for TAp73 in driving the pentose phosphate pathway to support cellular proliferation, and these E1a/Ras-transformed TAp73^−/−^ MEFs also developed smaller tumors in xenograft models (9). These results appear to be in agreement with those of the study that suggests a proangiogenic role for TAp73 (13), in which *p53* mutations were excluded by direct sequencing of the cell lines used (data not shown) (Table 1). Thus, the *p53* status of the E1a/Ras-transformed TAp73^−/−^ MEFs used in the study that found them to form larger tumors and thus have enhanced angiogenesis could shed light and be informative. Using the Eμ-Myc model, earlier studies showed that the lack of both p73 isoforms had negligible effects on disease onset and overall survival (24), whereas the recent observations indicated that TAp73's absence promoted angiogenesis, insinuating enhanced tumor predisposition (12). In the former case, *p53* was found not to be mutated, but the status of *p53* was not reported in the latter. Given that mutant p53 can have novel gains of function that drive multiple oncogenic processes (25), the presence of mutated p53 may not be equivalent to its total loss or inactivation by oncogenes and may influence cellular outcomes. Thus, a different *p53* status may potentially also contribute to a different outcome of TAp73's effect on angiogenesis and requires further investigation.

## THE COLLECTIVE MODEL: A BIFUNCTIONAL ROLE FOR TAp73 IN ANGIOGENESIS

**FIG 1:**
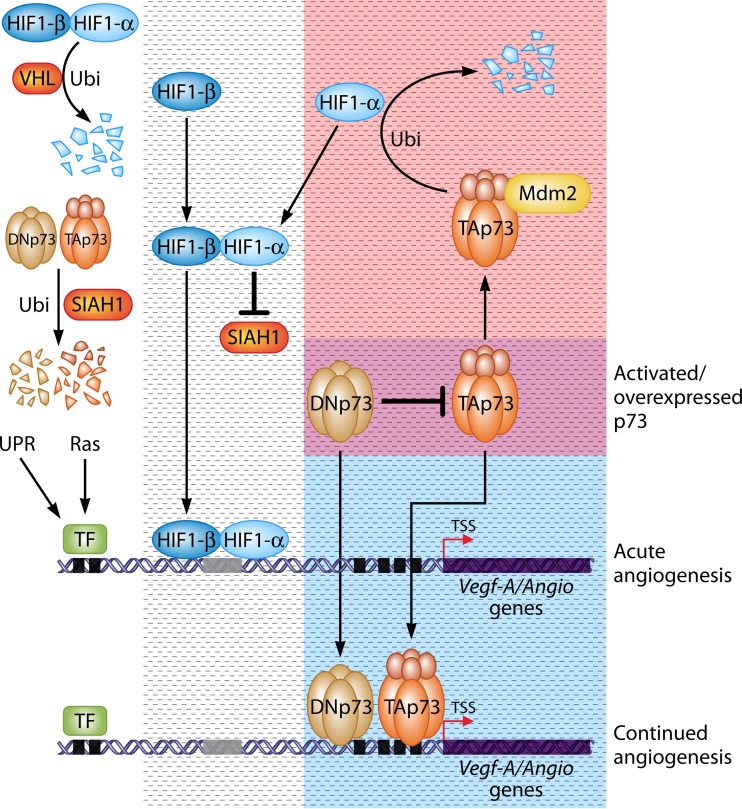
Model for TAp73s's bifunctional role in regulating angiogenesis. In normoxic conditions (white open area), HIF1-α is unstable, being kept in check by the E3 ligase VHL. Independently, the E3 ligase SIAH1 keeps TAp73 and DNp73 levels in check by promoting their degradation through ubiquitination. Upon hypoxia (dashed box), HIF1-α is stabilized through relief from VHL, dimerizes with HIF1-β, and goes on to turn on its canonical angiogenic target genes, including *Vegf-A*, through direct binding of the HREs (represented by the gray rectangle in the gene structure), resulting in the acute effects of angiogenesis. Concurrently, HIF1-α causes the suppression of SIAH1, thereby relieving TAp73/DNp73 degradation and leading to stabilization of the latter. TAp73 and DNp73 then go on to directly bind to other promoter regions (represented by black squares) on the *Vegf-A*/angiogenic target genes, turning on their expression independent of the requirement for HRE. This could potentially constitute a secondary amplification event in response to hypoxia, leading to continuous angiogenesis independent of HIF-1α, as has been shown with the unfolded protein response (UPR) or by Ras activation. This scenario could also occur in the tumor context when TAp73 or DNp73 is overexpressed (blue box, reflecting a subset of the hypoxic response), regardless of the oxygen tension, altogether resulting in the positive effect of hypoxia or the oncogenic-state-dependent, TAp73/DNp73-mediated regulation of angiogenesis. In contrast, TAp73 can also bind to MDM2, thereby recruiting it to cause the degradation of HIF1-α, which thus abrogates HIF1-α-mediated angiogenesis. This could be a mechanism that operates during hypoxic conditions to switch off the hypoxic response, thereby denoting a negative regulatory loop to shut down HIF1-α. Alternatively, one could envisage this scenario occurring in a tumor-suppressive context or in a state of exposure to genotoxic stress (pink box, reflecting a subset of the hypoxic response), whereby TAp73 is stabilized to exhibit its tumor-suppressive properties. The intersection of the blue and pink boxes (dark pink box) represents the activated state of TAp73 in response to either hypoxia or DNA damage or the state in cancers where the p73 proteins are overexpressed. A point of note is that DNp73 could also inhibit TAp73's ability to recruit MDM2 to degrade HIF1-α in this context, thus indirectly promoting angiogenesis. Further investigations are required to clarify the secondary wave of angiogenesis that is regulated by TAp73 during hypoxia and the contexts in which TAp73 acts as a show stopper to inhibit the hypoxic response. Other factors, such as *p53* status, may act as modifiers of the p73-mediated angiogenic response. The *Vegf-A* gene structure is shown as an exemplary angiogenic target gene. TSS, transcription start site; TF, transcription factors.

Besides the distinct variables that could act as determinants of TAp73's effect on angiogenesis, one could also view the published data in a collective model in which TAp73 can act as both a positive and negative regulator in different spatiotemporal contexts (Fig. 1). Upon hypoxia, HIF1-α is induced through the relief from VHL-mediated degradation (16), which then leads to the stabilization of TAp73 (and DNp73) via the suppression of SIAH1. While HIF1-α is the first step in directly turning on the angiogenic program to drive angiogenesis (26), its activation is required for stabilization of TAp73/DNp73, which then go on to transactivate the expression of angiogenic target genes without the requirement of HIF1-α. This may form part of a secondary wave to amplify and keep the angiogenic response turned on upon hypoxia in cases where p73 levels are low. Alternatively, this can be a phenomenon in cancers where the baseline TAp73 and DNp73 levels are constitutively high due to overexpression (Fig. 1, bottom right, blue box) (7, 8), thereby providing a survival signal and facilitating tumor growth. This possibility is not totally unexpected, as there are examples of HIF1-α independent regulation of *Vegf-A* and other angiogenic genes by K-Ras (27) or by ATF4 during the unfolded protein response (28), highlighting the existence of such scenarios. Upon cessation of the hypoxic response (e.g., after sufficient vasculature formation), the stabilized TAp73 may then be able to suppress HIF1-α expression through MDM2-mediated degradation, thereby turning off the HIF1-α-dependent angiogenic program. This regulation would imply the existence of a negative regulatory loop, akin to the p53-MDM2-p53 axis (29). Although this was not shown directly, an expectation is that TAp73 deficiency would lead to sustained HIF1-α activation and, thus, angiogenesis, which appears to be the case in one instance (12) but not in the other (13). Nonetheless, further evaluation is required to confirm this. Alternatively, TAp73 activation in tumor-suppressive contexts or genotoxic-stress contexts, such as upon exposure to DNA-damaging agents, would be able to suppress HIF1-α expression through MDM2-mediated degradation (Fig. 1, top right, red box). This would thus prevent the activation of the HIF1-α-dependent angiogenic program to suppress the growth of cells indirectly. As such a scenario would occur under nonhypoxic conditions, activation of the basal angiogenic program is possible in the absence of TAp73. A noteworthy point here is that HIF1-α is generally at very low levels in cells unless induced by hypoxic conditions, and thus, this scenario would require the suppression of the basal activity of HIF1-α by TAp73.

Nevertheless, these possibilities would require differing contexts in which both TAp73 and HIF1-α regulate each other's abundance through different E3 ligases, thereby having opposite effects on angiogenesis. In the cancer scenario, overexpressed TAp73 and DNp73 would favor a continued angiogenesis program that promotes tumor growth, even in the absence of hypoxia. This would also be the case under hypoxic and/or other growth-promoting conditions where both p73 proteins are stabilized to promote angiogenesis. In contrast, stress-mediated TAp73 activation would likely lead to suppression of the HIF1-α circuitry, thereby shutting down angiogenesis to enhance tumor suppression. Intriguingly, these possibilities indicate that the activation state of TAp73 may, in a spatiotemporal- and perhaps cell-type-specific manner, have a role in determining the outcomes. One could thus envisage that the cellular context could be dictating the functional interaction between TAp73 and the appropriate cofactors and DNA-modifying enzymes to ensure the specific cell fate outcomes, as has been shown with p53 in the context of selectivity in activating apoptosis or cell cycle arrest (30–32). Insofar as the role of TAp73 is concerned, it appears to be a critical regulator determining the angiogenic outcome based on the cellular context, thereby distinguishing itself from its homologue p53. How TAp73 acts as both a positive and negative regulator of angiogenesis is at present unclear. However, as mentioned above, given that TAp73 is activated both by DNA damage signals and growth factors, the cellular milieu will likely dictate TAp73's role in this cell fate decision with respect to angiogenesis. On the other hand, the oncogenic DNp73 plays its role in promoting angiogenesis either directly (14) or indirectly, by inhibiting TAp73 in its negative regulation of HIF1-α (12). Nevertheless, the p73 aficionados would have to work out why DNp73 is not acting in a dominant-negative manner in the former context.

## CONCLUSIONS

In summary, the p73 proteins have been shown to regulate angiogenesis, with the antiapoptotic DNp73 form having a clear role in promoting this phenomenon, whereas the tumor-suppressive TAp73 has both a positive and a negative effect, reflecting its dual nature of promoting or inhibiting cellular growth, and thus, tumorigenesis. The circumstances in which TAp73 exhibits its opposite functions could be dependent on the strength and the spatiotemporal context of its activation, either in the hypoxic context to initiate and terminate the signaling cascade or in different contexts of cellular stress, and could also be further influenced by other modifiers, such as the status of *p53*. Future work will shed light to answer some of the questions raised here and, thus, provide detailed mechanistic insights into the contextual operation of TAp73 in regulating angiogenesis, both positively and negatively.
